# Safety of mRNA COVID-19 Vaccines in Patients with Inborn Errors of Immunity: an Italian Multicentric Study

**DOI:** 10.1007/s10875-022-01402-6

**Published:** 2022-11-14

**Authors:** Cinzia Milito, Francesco Cinetto, Giulia Garzi, Andrea Palladino, Marco Puca, Elena Brambilla, Camilla De Vitis, Giulia Costanzo, Riccardo Scarpa, Alessandra Punziano, Gianluca Lagnese, Stefano Del Giacco, Giuseppe Spadaro, Isabella Quinti, Davide Firinu

**Affiliations:** 1 Department of Molecular Medicine, Sapienza University of Rome, Rome, Italy; 2 Department of Medicine-DIMED, University of Padova, Padua, Italy; 3 Rare Diseases Referral Center, Internal Medicine I, Ca’ Foncello Hospital, AULSS2 Marca Trevigiana, Treviso, Italy; 4 Department of Medical Sciences and Public Health, University of Cagliari, Azienda Ospedaliero Universitaria, SS 554-Bivio Sestu, 09042 Monserrato, CA Italy; 5 Department of Translational Medical Sciences, University of Naples Federico II, Naples, Italy

**Keywords:** Vaccines, COVID-19, IEI patients, adverse reaction, safety profile, CVID, immunodeficiency

## Abstract

**Purpose:**

Little is known about vaccine safety in inborn errors of immunity (IEI) patients during the current vaccination campaign for COVID-19. To better investigate the reactogenicity and adverse event profile after two, three, and four doses of mRNA vaccines, we conducted an observational, multicentric study on 342 PID patients from four Italian Referral Centres.

**Methods:**

We conducted a survey on self-reported adverse reactions in IEI patients who received mRNA vaccine by administering a questionnaire after each dose.

**Results:**

Over the whole study period, none of the patients needed hospitalization or had hypersensitivity reactions, including anaphylaxis and delayed injection site reaction. After two vaccination doses, 35.4% of patients showed only local reactogenicity-related symptoms (RrS), 44.4% reported both systemic and local RrS, and 5% reported only systemic RrS. In more than 60% of cases, local or systemic RrS were mild. After the first and second booster doses, patients showed fewer adverse events (AEs) than after the first vaccination course. Patients aged 50 years and older reported adverse events and RrS less frequently. Among AEs requiring treatment, one common variable immune deficiency patient affected by T cell large granular lymphocytic leukemia developed neutropenia and one patient had Bell’s paralysis perhaps during herpes zoster reactivation.

**Conclusion:**

Although our follow-up period is relatively short, the safety data we reported are reassuring. This data would help to contrast the vaccine hesitancy often manifested by patients with IEI and to better inform their healthcare providers.

**Supplementary Information:**

The online version contains supplementary material available at 10.1007/s10875-022-01402-6.

## Introduction

Coronavirus disease (COVID-19), first reported in China on late December 2019, has rapidly expanded globally becoming a pandemic. The World Health Organization encourages vaccination against COVID-19 as a measure to contain the pandemic [1, 2].

To date, five COVID-19 vaccines have been approved in Italy: the mRNA-based BNT162b2 (Pfizer-BioNTech) and the mRNA-1273 (Moderna), since late December 2020; the viral vector–based vaccines ChAdOx1 nCoV-19 (Oxford-AstraZeneca) and the Janssen (Johnson&Johnson), since late January and March 2021 respectively; and finally, the Nuvaxovid (Novavax), based on protein subunits, authorized on December 22, 2021 [3, 4].

In Italy, a national vaccination program was started in late December 2020 including primarily healthcare workers, the older population, and people living with severe chronic diseases. Subsequently, the program has been expanded to other categories of patients and to the general population and is currently ongoing. Currently, the fourth vaccine dose has been approved only for people > 65 years and for high-risk categories such as subjects with increased infectious risk, due to oncological or immunosuppressive therapies, organ transplantation, and primary immunodeficiencies [5].

Patients with inborn errors of immunity (IEI) have been prioritized and started being vaccinated since January 2021 with mRNA vaccines [6]. In Italy, mass vaccination has mainly been carried out using the BNT162b2 and mRNA-1273 vaccines, currently approved for the booster and second booster (fourth) dose.

Up to now, the published literature about IEI patients is mainly focused about vaccines’ immunogenicity. Patients with mild antibody deficiencies, patients with phagocyte defects, and patients with combined B and T cell immunodeficiency and uncomplicated common variable immune deficiency (CVID) showed different seroconversion rates with a good antibody and T cell response, whereas patients with X-linked agammaglobulinemia and complicated CVID with non-infectious comorbidities had a poor response. In patients with a cellular immunity defect, an adequate seroconversion was observed in 50 to 80% after the administration of 2 or 3 mRNA vaccine doses [5, 7, 8], whereas in IEI patients with predominant antibody deficiency (PAD), a seroconversion rate of about 33% after two doses and about 60% upon three doses has been observed previous studies [9, 10].

Moreover, the studies that assessed the antibody-neutralizing activity showed that only a limited number of CVID patients can develop virus-neutralizing antibodies [8, 11]. Overall, these data suggest that both the quantity and quality of the humoral immune response to COVID-19 vaccination may be suboptimal in CVID subjects [10]. Recent studies have also shown that in CVID patients immunization with two doses of mRNA vaccine did not generate spike-specific classical memory B cells (mB) but atypical mB with low binding capacity to spike protein without receptor binding domain–positive memory B cells [12], indicating a different capability of B cells to undergo somatic mutation and affinity maturation in the germinal centers which are indispensable for the establishment of long-term immunity [10]. Spike-specific T cell responses were induced with a variable frequency.

In terms of safety, the clinical trials for registration of mRNA-based vaccines involved the general population and local and systemic adverse reactions have been described. The incidence of adverse events (AEs) was significantly higher in younger participants than in older ones, in females than in males, and after the second dose than after the first dose. Limited data is available about AEs after third and fourth vaccine doses [13, 14].

Conversely, few data has been reported about vaccine safety in IEI patients, especially focusing on severe adverse events (SAE). In a paper published on a Dutch cohort, 1.8% (9/505) of immunized patients reported the occurrence of adverse events including breath shortness, dyspnea and low oxygen levels, cerebral hemorrhage, thrombosis, and diverticulitis [15].

Data about safety profile of COVID-19 vaccines, including reactogenicity and other adverse drug reactions (ADRs), in IEI patients may be useful in the management of these patients, also providing data to face vaccine hesitancy [16].

To better investigate and define the impact of AEs after two, three, and four doses of COVID-19 vaccine in a cohort of IEI patients, we conducted an observational, multicentric study involving 342 PID patients from four Italian Referral Centres for Primary Immunodeficiency (Rome, Naples, Padua and Cagliari). Therefore, we conducted a survey on self-reported adverse reactions in IEI patients who received either the BNT162b2 or the mRNA-1273 vaccine by administering a questionnaire after each vaccine dose.

## Materials and Methods

### Study Design

A questionnaire survey was conducted by phone or during a medical examination by the medical staff of involved centers within 7 days after each vaccine dose administration, re-evaluating over time the duration and evolution of adverse events. We analyzed cumulative data of AEs reported after the first and second vaccine doses, considering that as the “vaccination cycle.” Then, we collected similar data through the same interview after the first and the second booster doses. The data of all subjects who had received two, three, or four doses of either the BNT162b2 vaccine or the mRNA1273 vaccine was selected for analysis. Only a very small proportion of patients received the ChAdOx1 nCoV-19 vaccine: we excluded these patients from the analysis.

For each patient enrolled in the study, we collected the following: the category of IEI; demographic and clinical variables, including sex, age, date, and type of vaccine; type of adverse reaction (distinguishing reactions related to reactogenicity, local or systemic, and other reactions not related to it), including the date of onset, duration, and severity of the adverse reactions. Local adverse events included pain, erythema, or swelling at the injection site. Systemic adverse events included fever, fatigue, headache, muscle and joint pain, nausea, diarrhea, and systemic skin rash. AEs not related to reactogenicity included hypersensitivity reactions, such as anaphylaxis, urticaria, and mouth or throat itching or tingling, heart palpitation, dizziness, and other uncommon ADRs.

The severity of adverse reactions was divided into three categories: mild (not requiring drugs and not interfering with daily routine activities), moderate (requiring treatment and causing difficulty with daily activities), and severe (causing loss of more than 1 day of work or requiring emergency room access or life-saving medications). The duration of the adverse reactions was also assessed. Delayed injection site reaction was defined as a skin reaction such as erythema or pruritus around the injection site, with an onset 48 h after vaccination. Information about delayed injection site reaction was obtained from responses to an open-ended question about other symptoms. We also investigated if AEs required the use of NSAIDs or other drugs or self-resolved without therapy.

The study has been approved by the Ethical Committee of the Sapienza University of Rome. Informed consent was waived by the Institutional Review Board because of the observational nature of the study and because participant identifiers were completely encrypted before analysis. The study was performed in accordance with the Good Clinical Practice guidelines, the International Conference on Harmonization guidelines, and the most recent version of the Declaration of Helsinki.

### Statistical Analysis

Categorical variables were reported as numbers (percentages) and the chi-square test was used to assess the statistical significance of differences between groups. All *p* values were two-sided, and *p* values < 0.05 were considered statistically significant.

## Results

### First Vaccination Cycle (Two Doses)

#### Enrolled Population

The cohort of patients included the following: 286 patients with common variable immunodeficiency (CVID), 23 patients with DiGeorge’s syndrome, 10 patients with X-linked agammaglobulinemia (XLA), 3 patients with selective Ig deficiency (including IgG subclass deficiency, selective IgA deficiency), 5 patients with hyperIgE syndrome (HIES), 3 patients with Good’s syndrome, 2 patients with autoimmune lymphoproliferative syndrome (ALPS), 2 patients with activated PI3 kinase delta syndrome (APDS), and 2 patients with agranulocytosis and other diseases like Kabuki syndrome (1), deficiency of adenosine deaminase 2 (DADA2, 1), immune-dysregulation with polyendocrinopathy and enteropathy, X-linked (IPEX, 1), Mendelian susceptibility to mycobacterial disease (MSMD, 1), and ataxia-telangiectasia (AT, 1). Characteristics of the enrolled population are resumed in Table 1.

**Table 1:** Demographic characteristics of enrolled population for each vaccination cycle

	1st vaccination cycle	Booster dose	2nd booster
Enrolled patients	342	266	61
Ataxia-telangiectasia	1	1	0
ALPS	2	1	1
APDS and APDS2	2	2	0
CMC	1	1	0
CVID	286	239	55
DADA2	1	0	0
DiGeorge syndrome	23	2	1
Good’s syndrome	3	3	1
HIES	5	3	0
Selective Ig deficiency	3	1	1
Kabuki syndrome	1	1	0
XLA	10	10	2
Agranulocytosis	2	0	0
IPEX	1	1	0
MSMD	1	1	0

*Legend*: *ALPS*, autoimmune lymphoproliferative syndrome; *APDS*, activated PI3K delta syndrome; *APDS2*, activated PI3K delta syndrome2; *CMC*, chronic mucocutaneous candidiasis; *CVID*, common variable immunodeficiency; *DADA2*, adenosine deaminases deficiency 2; *HIES*, hyper IgE syndrome; *XLA*, X-linked agammaglobulinemia; *IPEX*, immunodeficiency, polyendocrinopathy, and enteropathy X-linked syndrome; *MSMD*, Mendelian susceptibility to mycobacterial diseases

Patients’ age range is between 17 and 86, with a median age of 48 years. The age group representation was as follows: 0–17 years old 0.3%, 18–49 years old 52.2%, 50–64 years old 27.6%, 65–74 years old 14.3%, 75–84 years old 5.2%, > 85 years old 0.3%. A total of 164 patients were male (48%) and 178 were female (52%).

#### Reported Adverse Events upon Two Vaccination Doses

In our cohort, 315 patients received BNT162b2 vaccine, and 25 received mRNA1273 (two vaccination doses 21 to 28 days apart).

None of the patients needed hospitalization or had hypersensitivity reactions, including anaphylaxis and delayed injection site reaction. One-hundred twenty-one patients (35.4%) showed only local reactogenicity-related symptoms (RrS), 152 patients (44.4%) reported both systemic and local RrS, and 17 patients (5%) reported only systemic RrS. Fifty-two patients (15.2%) did not show any AE. Other ADRs have been reported to be only associated with at least one RrS in 21 patients (6.1%) and in 75% were mild (Table 2). Local RrS were mild in 67.6%, moderate in 28%, and severe in 4.4% of cases. Systemic RrS were mild in 60.7%, moderate in 34%, and severe in 5.3% of cases. More data about frequency and severity of single types of reported AEs is reported in the Supplementary Materials.

**Table 2:** Type of AEs and patients involved

	1st vaccination cycle (342 pt)		Booster (266 pt)		2nd booster (61 pt)	
	*N*	%	*N*	%	*N*	%
Local AEs	121	35.38%	77	28.95%	12	19.67%
Systemic AEs	17	4.97%	25	9.40%	5	8.20%
Local + systemic AEs	152	44.44%	90	33.83%	14	22.95%
No ADRs	52	15.20%	74	27.82%	30	49.18%
Non RrS ADRs	21	6.14%	21	7.89%	2	3.28%

*Legend*: *AEs*, adverse events; *ADRs*, adverse drug reactions; *RrS*, reactogenicity-related symptoms

Two patients showed local lymphadenopathy involving the injection site arm and/or the homolateral cervical lymph node chain. One patient complained of cough, rhinitis, and pharyngodynia. Two patients had arm or leg paraesthesia and one reported trigeminal neuralgia, after the first vaccine dose. In particular, the trigeminal neuralgia subsequently extended to the whole hemilatum of the head and was diagnosed as Bell’s palsy. It occurred in a CVID patient about 48 h after the injection of the first dose of BNT162b2 vaccine. The patient had a previous herpes zoster infection at the same site of the face and it was treated for 7 days with acyclovir, paracetamol, and NSAIDs, with resolution of symptoms within 2 weeks. One patient affected by IgA and IgG subclass deficiency showed severe neutropenia 3 days after the first dose administration of BNT162b2, which was treated with granulocyte stimulating factor, oral corticosteroid therapy, and antibiotics for 5 days until resolution.

Comparing the group of patients who received BNT162b2 and the group who received mRNA1273, we did not observe substantial differences in safety profile, though a low prevalence of AEs (fever) has been reported in patients who received mRNA-1273 vaccine (fever 16.2% of patients vaccinated with BNT162b2 vs 28% with mRNA1273). The mean duration of symptoms was 1.8 days and 76.3% of the patients did not take any drug. NSAIDs, in particular ketoprofen and ibuprofen, were the most used treatment (17%). Paracetamol was used by 5.8% of symptomatic patients. One patient took antihistamine medication for mild itching at the injection site.

Upon two vaccination doses, the number of self-reported adverse events has been significatively higher in females with 488 AEs (including pain, swelling, redness and itching on the site of inoculation, fatigue, headache, chills, joint pain, muscle aches, nausea, vomiting, diarrhea, and abdominal pain) vs 305 AEs in males (*p* = 0.0001). Fever was reported by 21 male patients vs 38 female patients (*p* = 0.0447).

### Booster Dose

#### Enrolled Population

We submitted the questionnaire to 314 patients, as 28 patients were not available or refused to participate at follow-up. After the first vaccination cycle, a total of 47 (15%) patients refused the vaccination or got infected. Two hundred sixty-six patients (77.8% of the total cohort) received the booster dose during the study time, including 235 patients with CVID, 10 patients with XLA, 2 patients with DiGeorge’s syndrome, 3 patients with HIES, one patient with selective Ig deficiency, 2 patients with Good’s syndrome, one patient with ALPS, 2 patients with APDS, and 1 patient with MSMD (Table 1). Patients’ age range is between 18 and 84, with a median age of 50 years. The age group representation was as follows: 18–49 years old 47.5%, 50–64 years old 28.7%, 65–74 years old 16.1%, 75–84 years old 5.4%. A total of 125 patients were male (47%) and 141 were female (53%). Two hundred and forty-three patients were re-vaccinated with BNT162b2 and 23 with mRNA1273, after a median time of 192 days (IQR 165–221) since the first vaccination cycle. ChAdOx1 nCoV-19 did not obtain approval for booster dose for fragile patients.

#### Reported Adverse Events upon Booster Vaccination Dose

After the booster dose, we observed a lower number of adverse events in comparison to the previous doses with a higher prevalence of mild symptoms. No patient needed to access the emergency room or used life-saving drugs and no hypersensitivity reaction has been reported.

Seventy-seven patients (29%) showed only local RrS of mild severity in 84.6% of cases, 90 patients (33.8%) reported both systemic and local RrS, and 25 patients (9.4%) reported only systemic RrS that were mild in 66.7% of cases, moderate in 34%, and severe in 5.3%, while 74 (27.8%) did not show any AE. Other ADRs have been reported to be only associated with at least one RrS in 21 patients (7.9%). Other ADRs, never reported singularly, were mild in 84.2% and moderate in 15.8% of cases (Table 2). The analysis of single AE frequency and severity is reported in the Supplementary Materials.

Three patients reported local lymphadenopathy involving the injection site arm and/or the homolateral lateral cervical lymph node chain or submandibular lymph node stations; two patients reported local paraesthesia and neuralgia in the legs or the arms. Other infrequent effects have been reported: one patient related dyspnea, one anosmia, one epigastric pain, one insomnia.

Overall, the median duration of the symptoms was 1.8 days. Similarly at the first two doses, patients used mostly NSAIDs (15.7% of cases) or paracetamol (8.4%) in order to relieve symptoms. One patient took antihistamine medication for mild itching at the injection site. One patient took oral corticosteroids for moderate flu-like symptoms.

Upon the third vaccination, the number of self-reported adverse events was significatively higher in female with 319 AEs (including pain, swelling, redness and itching on the site of inoculation, fatigue, headache, chills, joint pain, muscle aches, nausea, vomiting, diarrhea, and abdominal pain) vs 170 AEs in male sex (*p* = 0.0002). Fever was reported by 16 male patients vs 30 female patients (*p* = 0.1346).

### Second Booster Vaccine Dose (Fourth)

#### Enrolled Population

We submitted the questionnaire to 119 patients. During the study time, 58 patients got infected meanwhile and a total of 61 patients (17.84% of the total cohort) received the fourth vaccine dose, including 55 patients with CVID and 6 affected by other PIDs (2 XLA, 1 DiGeorge’s syndrome, 1 ALPS, 1 Good’s syndrome, and 1 patient with selective IgA deficiency) (Table 1). Sixty patients received BNT162b2 and just one CVID patient received mRNA1273, after a median time of 159 days (IQR 145–170). Patients’ age range is between 18 and 84, with a median age of 52 years. The age group representation was as follows: 18–49 years old 44.06%, 50–64 years old 27.1%, 65–74 years old 23.7%, 75–84 years old 3.3%. Twenty-seven patients were male (44.3%) and 34 were female (55.7%).

#### Reported Adverse Events upon Second Booster Vaccination Dose

Cumulative self-reported AEs have been recorded by 41.4% of patients, almost exclusively of mild severity, and no severe AEs have been registered. Also in this case, no hypersensitivity reactions have been reported.

Twelve patients (19.7%) showed only local RrS almost totally mild (90%), and 14 patients (23%) reported both systemic and local RrS. Five patients (8.2%) reported only systemic RrS, mild in 86.8% of cases. Thirty (49.2%) did not show any AE. Other ADRs have been reported to be only associated with at least one RrS in 2 patients (3.3%). Other ADRs, exclusively mild, have never been reported singularly (Table 2). More data about single AE frequency and severity is reported in the Supplementary Materials. The mean of the duration of symptoms was 1.4 days. Few patients used drugs to relieve the symptoms, mostly NSAIDs or paracetamol (10.3% in both cases). One patient took antibiotic therapy by himself for fever (> 38.5 °C).

Also, upon the fourth vaccination dose, the number of self-reported adverse events was significatively higher in female sex with 71 AE (including pain, swelling, redness and itching on the site of inoculation, fatigue, headache, chills, joint pain, muscle aches, nausea, vomiting, diarrhea, and abdominal pain) vs 27 AE in male sex (*p* = 0.0017). Fever was reported only by 5 female patients (*p* = 0.0662).

### Reported Adverse Events Sorted by Age

Analyzing data by age groups, and in particular dividing patients over and under the age of 50, there was no statistically significant difference in self-reported AE (of any severity degree) upon the first vaccine cycle (*p* = 0.33) and four doses of vaccine (*p* = 0.1748).

On the other hand, we found an important difference in self-reported AE between age groups after booster dose (*p* = 0.039) with higher number of AE in younger patients compared to older ones.

Regarding fever, we did not find any differences between age groups upon two (*p* = 0.77), three (*p* = 0.62), and four doses of vaccine (*p* = 0.37).

## Discussion

There are currently few published studies on post-vaccination adverse events in patients with primary immunodeficiency, probably because it is a group of rare diseases and few affected individuals have been vaccinated in relation to the general population. Since most of the enrolled patients are affected by CVID and considering that CVID has an estimated prevalence of 1:50,000–1:25,000, the population enrolled in this study likely represents the 10–20% of the Italian CVID population [17].

In a recent Swedish trial on the safety and efficacy of BNT162b2 vaccine in five groups of immunocompromised patients (including 90 PID subjects), a higher rate of systemic reactogenicity was observed among healthy controls than in the patient group after the second dose [18], and this has been linked to immunosuppression. Since it was reported that most AEs occurred among allogeneic HSCT/CAR-T cell–treated patients, followed by patients with PID and those with solid organ transplantation, the correlation of AEs with the severity of the immune deficiency appeared to be directly related.

We found that the reactogenicity in the PID cohort was overall lower than that of clinical trials data and from early post-authorization monitoring studies [19, 20], similar or reduced to that reported in observational or real-world studies after two doses [14, 21], and comparable or slightly reduced after boosters as well [22–24].

In fact, we found that the IEI participants more frequently reported transient reactions of mild severity, with a short duration and a little impact on daily activities and a low use of medications. This is in line with other reports about 2 doses in IEI [5, 11, 25–27].

Delmonte et al. recently reported about 81 patients with IEI who underwent first vaccination cycle with BNT162b2, testing humoral response in comparison with healthy controls. In the IEI group, they mainly observed the occurrence of local reactions such as pain at the injection site, and systemic AEs were mostly reported after the administration of the second dose [28].

IEI patients show a variably reduced response to vaccinations [25, 29, 30] in terms of antibody production and specific immune cell activation and we may speculate that they probably also exhibit fewer symptoms and adverse events than the general population, due to their immune underlying defect. This is also highlighted by a recent Hungarian study, testing specific antibody and the T cell response elicited by BNT162b2 boosting after two ChAdOx1 shots in a cohort of CVID patients, compared with healthy control subjects. The authors also reported the AE profile, showing that CVID patients who had a worse immune response to vaccinations might have a slightly reduced rate of systemic side effects [31].

Female participants reported adverse events and reactions more frequently than male participants, as reported in other studies [18].

In our cohort, above all upon the booster dose, patients aged 50 years and older reported adverse events and reactions less frequently than the younger ones, in accordance with what is shown in other published works, regarding healthy population.

Overall, after the booster dose our patients showed fewer AEs than after the first vaccination course. Moreover, self-reported AEs were for the most part mild, highlighting a good safety profile of the mRNA vaccines, most commonly used [32].

Reports about the measures of health impact, although self-assessed and subjective, correlate with reports about reactogenicity: in our cohort, more health impacts were reported by female than by male recipients, and effects are more frequently reported after dose two compared with booster dose. Furthermore, a larger number of AE have been reported in our study by those who received mRNA-1273 versus BNT162b2, although there is an important difference in the proportion of IEI patients being vaccinated with BNT162b2 and mRNA1273 both for first vaccination schedule and for the first and second booster doses. This evidence is supported by other papers about pharmacovigilance and clinical trials, in which it is highlighted that a slightly greater adverse effect was reported with mRNA1273, although less frequent effects such as insomnia, hyperhidrosis, diarrhea, and loss of appetite are reported with BNT162b2. Lymphadenopathy and delayed injection site reactions are more frequently reported with mRNA1273.

In a BNT162b2 phase I–II trial, a transient neutropenia has been very rarely described 6–8 days after the second dose without clinical manifestations [33], and another study did not find an association between risk of neutropenia and BNT162b2 [34]. A transient neutropenia has also been reported in a few cases in immunocompromised patients, receiving BNT162b2 [18]. In our patients, neutropenia occurred in a patient with CVID and required treatment. The patient was later diagnosed also with T cell large granular lymphocytic leukemia stage 4b, which includes neutropenia among its manifestations. Thus, a possible role of this comorbidity should be considered.

In terms of the types of reactions manifested, nonspecific post-vaccination adverse effects were prevalent, such as reported by other works. In particular, the most frequent AEs in the general population were injection site pain, fever, headache, and muscle aches. On the other side, in our cohort fatigue seems to be most frequently reported and quite frequent as in clinical trials [19, 35]. This is probably linked to the fact IEI patients are highly medicalized and affected by chronic diseases, and this may influence side effect report [35]. Similarly, a higher frequency of asthenia and muscle pain was reported in cohorts of oncological patients in active treatment compared to healthy controls or subjects with oncological disease not actively treated [36].

Common symptoms, as for the general population, include fever, myalgias, and fatigue, but severity did not increase in our cohort of patients with IEI. In patients with IEI complicated by autoimmunity or primary immune-dysregulation disorders, vaccinations have been similarly well tolerated without worsening of autoimmune conditions [25, 37].

Rare severe AEs, such as myocarditis and ITP, have not been reported in our cohort, as well as anaphylaxis.

In the general population, the myocarditis reporting rates were 40.6 and 2.4 cases per million doses in males aged 12 − 29 and ≥ 30 years, respectively, and 4.2 and 1.0 cases per million doses in females aged 12 − 29 and ≥ 30 years, respectively [38]. In a population of patients affected by secondary immunodeficiency, the incidence was similar to the general population [39].

One explanation for myocarditis following mRNA vaccine immunization is that exogenous mRNA may potentially act as an antigen and activates inflammatory pathways. Another explanation is the cross-reaction between the anti-S-protein antibodies and self-antigens [40]. All these immune mechanisms are variably deficient in patients with IEI, so they may be partially protected against this kind of immune-mediated reactions. This could be hypothesized also for anaphylaxis: PID patients often exhibit absent to low IgE levels and lack many immunological mechanisms that mediate immediate allergic reactions [41].

Bell’s palsy occurred in < 0.1% of the vaccine group and the placebo group (3 participants and 1 participant, respectively) during the observation period of the mRNA-1273 trial [27], and an increased risk has not been detected for BNT162b2 [42]. In a recent international survey about pediatric patients with MIS-C, it is reported that one 13-year-old male recipient was reported with acute-onset facial nerve palsy (Bell’s palsy) 1 week after his second BNT162b2 vaccine [43]. In our cohort, a patient had a neurological picture including Bell’s palsy following the first dose. This CVID patient with an inflammatory phenotype previously had a similar episode during herpes zoster reactivation. The real incidence of herpes zoster reactivation after COVID-19 vaccination is debated [44, 45], and our patient did not have skin eruption but responded to the antiviral treatment with full recovery.

There are also reports of the rare occurrence of post-vaccination ITP (de novo or relapse) in patients receiving mRNA vaccines (including BNT162b2) [46], but nation-wide studies did not confirm this observation [47, 48].

A variable proportion of CVID patients have ITP as an hematologic comorbidity in their medical history [49]. We did not observe de novo or relapses of ITP in the post-vaccine observation period, while for instance a relapse has been observed during SARS-CoV-2 infection in our PID cohort [50]. Since some AEs of vaccines such as ITP are immune-mediated, it is possible that use of immunoglobulin replacement therapy in PID patients may provide some risk reduction against these adverse effects by its immunomodulatory effect [51].

Although the follow-up period in our study is relatively short, the safety data that emerged is reassuring. This would help to contrast the vaccine hesitancy often manifested by patients with IEI and to better inform their healthcare providers [16]. This issue is also present in our cohort, as shown by the limited adherence to the second booster dose.

## Conclusions

This study has several limitations. First, given the nature of the self-reported survey, the frequency of reported adverse reactions may have been over- or underestimated and there may have been some recall bias. Second, most of the reported adverse reactions were not medically verified. Third, we evaluated only short/medium-term adverse events, and long-term surveillance is required to investigate possible long-term effects. However, it is a cohort of patients well-characterized and with an ongoing follow-up at our centers.

Furthermore, most patients received the BNT162b2 vaccine; hence, the results may not be generalizable to those experienced after other vaccines or their combinations, and the study population that underwent boosting was progressively reduced during the study period. This occurred, on the one hand, because many patients contracted the SARS-CoV2 infection and therefore postponed the vaccination, and on the other hand because some patients refused to continue vaccination beyond the primary vaccination course. Ultimately, some patients have planned their fourth dose in the coming weeks.

Data collection on fourth dose AEs and long-term monitoring in PID patients will continue in an ongoing prospective study about this topic.

## Supplementary Information

Below is the link to the electronic supplementary material.

Supplementary file1 (DOCX 13 KB)

## Data Availability

Data is available upon reasonable request to the corresponding author.
