# Supplementary material for: Safety of mRNA COVID-19 Vaccines in Patients with Inborn Errors of Immunity: an Italian Multicentric Study
Source: J Clin Immunol. 2022 Nov 14;43(2):299–307. doi: 10.1007/s10875-022-01402-6 (PMC9662105; doi:10.1007/s10875-022-01402-6)
Supplement: Supplementary file 1 — Supplementary file1 (DOCX 13 KB) [file 10875_2022_1402_MOESM1_ESM.docx]

**Supplementary materials for manuscript:**

Safety of COVID-19 vaccines and adverse events in IEI patients: an Italian multicentric study

First vaccination cycle reported AE:

We observed that the most frequent common symptom was pain at the site of inoculation (79.8% [50,9% mild, 25.1% moderate, 3.8% severe]), followed by fatigue (36.9% [19.3% mild, 15.8% moderate, 1.8% severe]), muscle aches (25,1% [17.5% mild, 6.1% moderate, 1.5% severe]), headache (22% [12.3% mild, 7.9% moderate, 1.8% severe]), joint pain (19.3% [10.8% mild, 7.3% moderate, 1.2% severe]), fever (17.25%) and chills (15.2% [8.5% mild, 5.8% moderate, 0.9% severe]). Other symptoms were reported in <10% of cases and were likely never severe.

AE reported after booster dose:

Injection site pain was reported in 61.3% of cases (52.1% mild, 9.2% moderate), fatigue in 32.2% (21.5% mild, 9.2% moderate, severe 1.5%), muscle aches in 18% (10.3% mild, 6.9% moderate, 0.8% severe), headache in 15.8% (10% mild, 5.4% moderate, 0.4% severe), fever in 17.2%, joint pain in 19.2% (12.3% mild, 6.1% moderate, 0.8% severe), chills in 11.1% (8.4% mild, 2.3% moderate). Other symptoms were reported in <5% of cases and were almost totally mild.

AE reported after second booster (fourth) dose:

Pain was present in 41.4% of cases (34.5% mild and 6.9% moderate), fatigue in 29.3% (24.1% mild and 5.2% moderate), headache in 18.9% (17.2% mild and 1.7% moderate), mild muscle aches in 15.5%, joint pain in 15.5% (13.8% mild, 1.7% moderate), fever in 8.6%. Mild redness and swelling at the injection site have been reported by 12% and 13.8% of patients, respectively. Other symptoms were reported in <5% of cases and were totally mild. In 58.6% of cases the patients reported no symptoms. Any uncommon AE has been reported.
